# Demographic aspects of human hydatidosis in Iranian general population based on serology: A systematic review and meta-analysis

**DOI:** 10.14202/vetworld.2018.1385-1396

**Published:** 2018-10-08

**Authors:** Shirzad Gholami, Asal Tanzifi, Mehdi Sharif, Ahmad Daryani, Mohammad-Taghi Rahimi, Siavash Mirshafiee, Shahabeddin Sarvi

**Affiliations:** 1 Toxoplasmosis Research Center, Mazandaran University of Medical Sciences, Sari, Iran; 2 Student Research Committee, Mazandaran University of Medical Sciences, Sari, Iran; 3 School of Medicine, Shahroud University of Medical Sciences, Shahroud, Iran; 4 Department of Husbandry, Ghaemshahr Branch of Islamic Azad University, Iran

**Keywords:** cystic echinococcosis, diagnosis, general population, hydatidosis, Iran, seroprevalence

## Abstract

**Aim:**

Human cystic echinococcosis (CE), caused by the larval stage of *Echinococcus granulosus* cestodes, is a globally distributed chronic disease that is an important socioeconomic and public health problem in humans and livestock in developing countries, including Iran. The aim of this study was to determine the overall seroprevalence of hydatid infection in the general population of Iran.

**Materials and Methods:**

This systematic review began by searching electronic databases in English (PubMed, Science Direct, Scopus, and Google Scholar) and Persian (Magiran, Scientific Information Database, Iran Medex, and Iran Doc).

**Results:**

Our search resulted in a total of 40 reports published from 1995 to 2015. Of 49,460 individuals surveyed, 3090 cases of hydatidosis were reported. Community-based studies showed that the seroprevalence of CE in the Iranian general population was 6.0% (95% confidence interval: 5.0-7.0%). The age group with the highest CE seroprevalence was 20-40 years, and the lowest one was in the under 20 year’s group. The seroprevalence of hydatidosis in males was significantly higher than that in females. In addition, the intended rate was significantly higher in rural regions than in urban areas.

**Conclusion:**

Management program for developing more efficient diagnostic tests should be established. Further, cost-effective preventive approaches, including relevant research, should be considered. Finally, hydatid cyst control programs that are important for interrupting the transmission of human CE should be improved.

## Introduction

Cystic echinococcosis (CE) or hydatidosis is a chronic disease caused by the larval stage of the *Echinococcus granulosus* parasite, a globally important helminth [1-3]. In addition to being a major public health problem in the world, many studies have shown that CE is an important socioeconomic concern. CE is recognized as an emerging or re-emerging disease, with a geographic distribution that is greater than previously recognized [3-6].

Humans acquire this infection by accidental ingestion of *E. granulosus* eggs with food, water, or contaminated soil. CE was included in the World Health Organization (WHO) initiative to assess the global burden of foodborne diseases [7]. The natural history of CE in humans usually includes several years of asymptomatic infection. The cysts are usually found in the liver (50-70%), lungs (20-30%), and, less commonly, in other organs (10%), for example, spleen, brain, kidneys, peritoneal cavity, muscle, bone, and heart [1,8,9]. Space in the body is occupied by hydatid cysts, and pressure on surrounding tissues typically causes clinical signs to develop. Anaphylactic shock and secondary CE are major complications caused by the rupture of cysts and spillage of their contents [2,10].

CE is a cosmopolitan zoonosis, with highly endemic areas in some regions of South America, North Africa, China, and the Middle East [2,10]. Iran is the only country in the Middle East where *Echinococcus* spp. has been found in natural host populations continuously to the present [11]. The previous studies revealed that Iran is one of the areas that has been known as hyperendemic area for CE by the WHO in terms of close relationship of a high proportion of society with animals, traditional animal husbandry, and then contact with the sources of infection, and 1% of all surgeries in this country can be attributed to CE [12-15].

Human cases of CE are regularly reported from medical centers in different parts of Iran, and the incidence of CE has been estimated at 1.18-3 per 100,000 populations [10,15,16]. Overall, the annual cost of CE is estimated at US $93.39 million for individuals living in Iran [10].

Some factors, such as exposure to contaminated soil, are closely linked to dogs, which play an essential role in the development and progression of CE [1,10]. However, many studies have examined the seroprevalence and effects of CE in Iran, and there is little information about the seroprevalence of *E. granulosus* infection in the general population. Therefore, the objective of the present meta-analysis was to estimate the seroprevalence of CE in the general population of Iran to evaluate the risk factors associated with this infection.

## Materials and Methods

### Ethical approval

This study is based on data and not on the animals so, ethical approval is not necessary to pursue such type of the study.

### Study design and data sources

Publications for the present systematic review and meta-analysis were collected from four English (PubMed, ScienceDirect, Scopus, and Google Scholar) and four Persian (Magiran, Scientific Information Database, Iran Medex, and Iran Doc) databases using the following search terms: “Hydatid cyst,” “*E. granulosus*,” “cystic echinococcosis,” “Iran,” “general population,” “serology,” “epidemiology,” “seroepidemiology,” and “seroprevalence.” Data were collected from a wide range of literature comprising full text articles, abstracts, and proceedings from national parasitological congresses in Iran which were published from 1995 to 2015.

### Study selection

To estimate the seroprevalence of CE in Iranian general population, cross-sectional studies were included in the analysis. CE was diagnosed in these studies by serological methods. Two researchers independently assessed studies for eligibility for inclusion in this analysis. Discrepancies between the researchers were resolved through discussion and consensus by a third reviewer for the accuracy and to remove conflict before starting of the study. Serological surveys carried out in other countries and studies that diagnosed infections with non-serological methods were excluded from the present study.

### Data extraction

In this review to provide comprehensive awareness, all studies that were based on serological methods and carried out to estimate the seroprevalence of CE in general populations in Iran were included. A data form was used to extract data consisting of the first author, year of publication, research locations, sample size, gender, and number of samples that were found positive for infection, age distribution, and methods. Information on risk factors including fruit and vegetable washing methods, contact with dogs, area of residence, education level, and occupation was also gathered.

### Statistical analysis

Since the wide variation was observed in included studies (Q=172.90, df=37, I^2^=98%, p<0.001), significant heterogeneity between studies was evident that is why we used to random effects instead of fixed effect. We calculated a pooled estimate of the prevalence (proportion) using a random effects model (reported as effect estimates with a 95% confidence interval [CI]). An overall seroprevalence and group-specific seroprevalences based on age (0-19, 20-40, 40-60, and ≥60 years), gender, and residential region were calculated. The heterogeneity among studies was evaluated (Der Simonian and Laird method) using the Cochran Q-test and I^2^ statistic. For the Q statistic, p<0.10 indicates statistically significant heterogeneity, and for the I^2^ statistic, I^2^ > 50% indicates a large degree of heterogeneity. A fixed effects model using the Mantel-Haenszel method was applied if the Q statistic was p<0.10 or I^2^ was >50%. The presence of heterogeneity was more through subgroups analysis and meta-regression. To evaluate the possibility of publication bias, an Egger weighted regression was performed. All statistical analyses were performed using Stata software version 11.0 (Stata Corp LP, College Station, TX, USA). p<0.05 was considered statistically significant.

## Results

**Figure-1:**
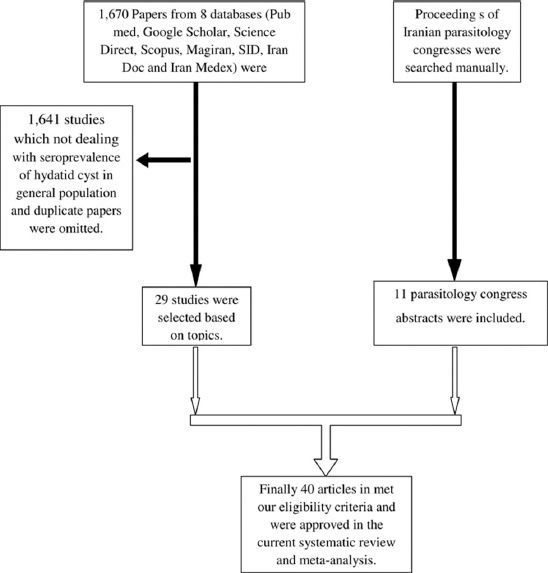
Flowchart describing the study design process.

**Figure-2:**
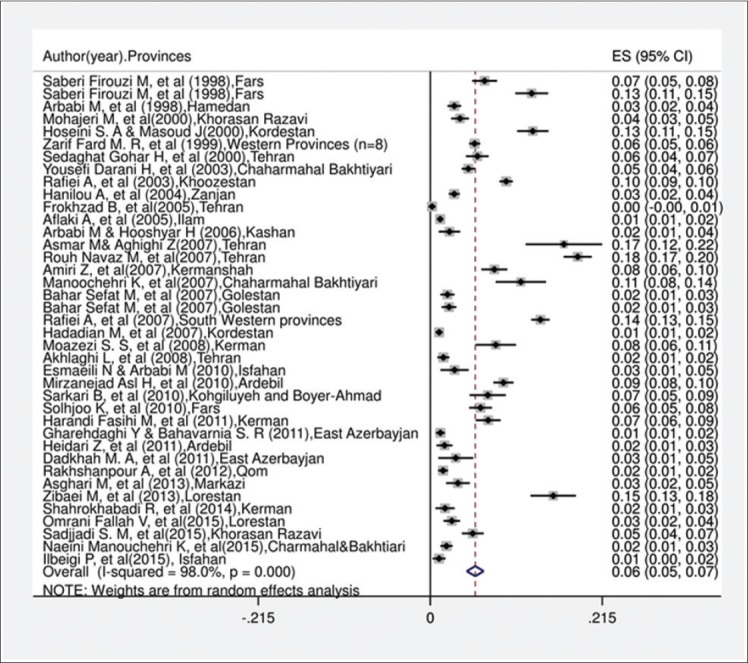
Forest plot for the prevalence of serology hydatidosis in general population in Iran.

**Figure-3:**
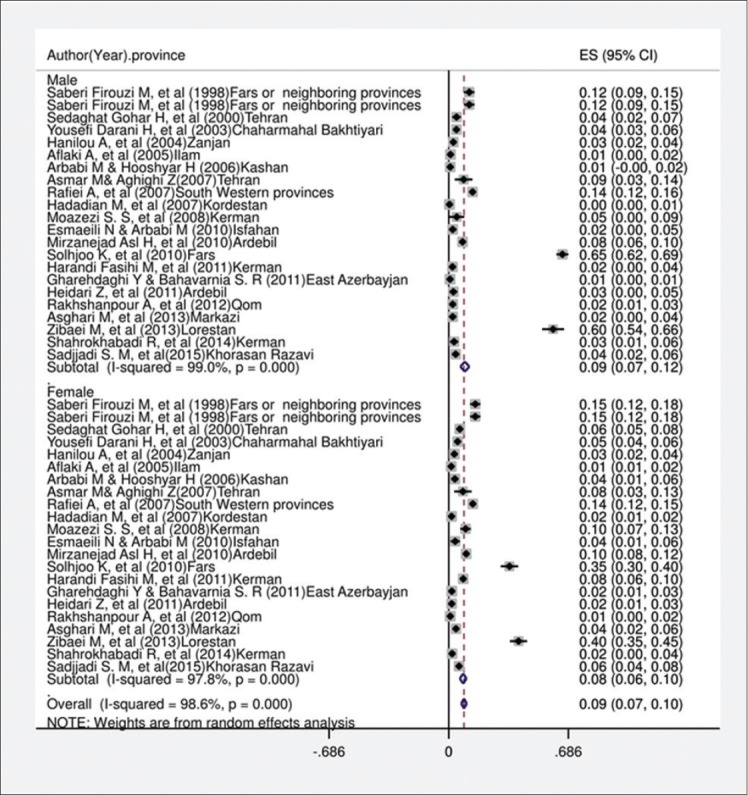
Forest plot for distribution seroprevalence of hydatidosis in male and female groups in Iran.

**Figure-4:**
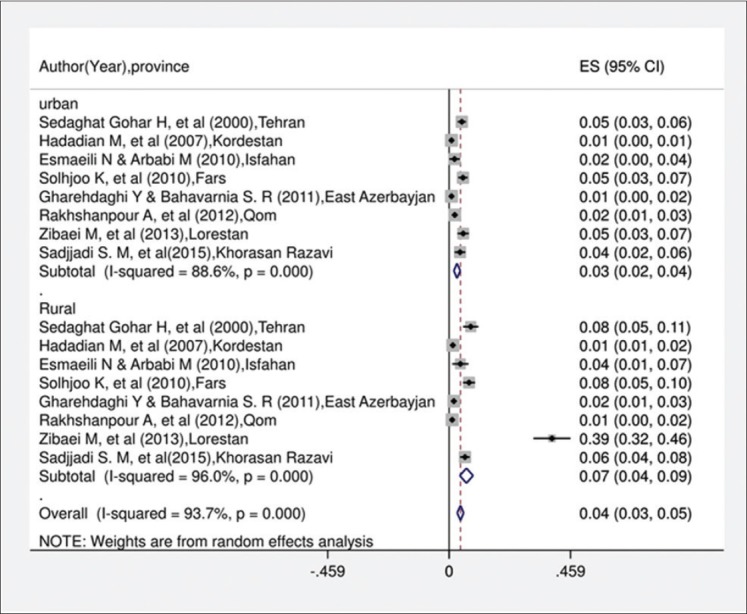
Forest plot for distribution seroprevalence of hydatidosis in urban and rural groups in Iran.

In the current systematic review and meta-analysis, of 1670 studies found in the literature search, 40 publications were included based on our criteria. Figure-1 shows a flowchart of the study design. Overall, 49,460 individuals, with 3090 seropositive cases, were included in the calculation of CE seroprevalence. The general characteristics and results of the studies included in this analysis are presented in Table-1[12,17-50]. To identify the sources of study heterogeneity, we performed a subgroup meta-analysis of five factors including mean age, sex, residential area, education level, and diagnostic test. There was wide variation in seroprevalence estimates across studies (Q=172.90, df=37, I^2^=98%, p<0.001). Using a random effects model, the seroprevalence of CE in the general population of Iran was found to be 6.0% (95% CI: 5.0-7.0%) (Figure-2). A subgroup analysis showed that the lowest and highest seroprevalences were in the age groups under 20 years (3.4%) and 20-40 years (10.6%) and that this difference was statistically significant. The seroprevalence of CE in males (9%; 95% CI: 7.0-12%) was significantly higher than that in females (8%; 95% CI: 6.0-10%) (z=51.02, df=1, p<0.001) (Figure-3 and Table-2). The seroprevalence of CE in rural regions (7.0%; 95% CI: 4.0-9.0%) was significantly higher than that in urban areas (3.0%; 95% CI: 2.0-4.0%) (z=3.90, df=1, p=0.048) (Figure-4 and Table-2).

**Table-1:** General characteristics of included studies in the current systematic review and meta-analysis.

Reference	Location (Province)	Lab method	Sample size	Positive	% (95% CI)	Gender		Residence	
	
						Male (%)	Female (%)	Urban (%)	Rural (%)
Saberi-Firouz *et al*. [20]	Fars	CIE	1000	68	6.8 (5.2-8.6)	11.80	15.20	-	-
Saberi-Firouz *et al*. [20]	Fars	ELISA	1000	127	12.7 (10.5-15.1)	11.80	15.20	-	-
Arbabi *et al*. [21]	Hamedan	IFA	1530	46	3 (2.2-4.1)	-	2.90	-	-
Zariffard *et al*. [22]	Western Provinces	ELISA	4138	230	5.5 (4.8-6.3)	4.70	6.20	4.60	7
Mohajeri *et al*. [23]	Khorasan Razavi	IFA	1100	41	3.7 (2.6-5.1)	-	-	-	-
Hosseini and Masoud [24]	Kordestan	IFA	1114	143	12.8 (10.8-15.1)	-	-	-	-
Sedaghat-Gohar [25]	Tehran	IFA	1052	62	5.8 (4.5-7.5)	4.50	6.36	4.80	8.10
Darani *et al*. [26]	Chaharmahal va Bakhtiari	CIE	2524	120	4.7 (3.9-5.6)	4.40	5.10	-	-
Hanilou *et al*. [27]	Zanjan	ELISA	2367	71	2.9 (2.3-3.7)	2.70	3.20	-	-
Aflaki *et al*. [28]	Ilam	Dot-ELISA	3000	37	1.2 (0.8-1.7)	1	1.47	0.56	12.34
Farokhzad *et al*. [29]	Tehran	IFA	437	1	0.2 (0.005-1.2)			-	-
Arbabi and Hooshyar [30]	Kashan	IHA	500	12	2.4 (1.2-4.1)	0.90	3.50	-	-
Asmar *et al*. [31]	Tehran	CIE	233	39	16.7 (11.9-22.8)	8.58	8.15	-	-
Rouhnavaz *et al*. [32]	Tehran	IFA	1842	340	18.4 (16.5-20.5)	-	-	-	-
Amiri *et al*. [33]	Kermanshah	IFA	1072	86	8 (6.4-9.9)	-	-	-	-
Manouchehri-Naeini *et al*. [34]	Chaharmahal va Bakhtiari	IHA	388	44	11.3 (8.2-15.2)	-	-	-	-
Baharsefat *et al*. [35]	Golestan	ELISA	1024	22	2.1 (1.3-3.2)	1.93	3.16	-	-
Baharsefat *et al*. [35]	Golestan	IFA	1024	24	2.3 (1.5-3.4)	-	-	2.47	2.45
Rafiei *et al*. [18]	South Western provinces	ELISA	3446	475	13.7 (12.5-15.1)	13.70	13.80	-	-
Hadadian *et al*. [36]	Kordestan	ELISA	1979	22	1.1 (0.6-1.6)	0.45	1.65	0.90	1.42
Moazezi *et al*. [37]	Kerman	ELISA	451	37	8.2 (5.7-11.3)	4.90	9.70	-	-
Tavalla *et al*. [19]	Tehran	Dot-ELISA	1100	18	16.3 (9.6-25.8)	-	-	-	-
Esmaeili and Arbabi [38]	Isfahan	IFA	361	11	3.1 (1.5-5.4)	2.30	3.70	2	4.2
Mirzanejad-Asl *et al*. [39]	Ardebil	ELISA	2008	184	9.1 (7.8-10.5)	7.90	10	-	-
Sarkari *et al*. [40]	Kohkiluyeh and Buyer Ahmad	ELISA	500	36	7.2 (5.1-9.9)	58.33	41.66	-	-
Solhjoo *et al*. [41]	Fars	ELISA	1096	69	6.2 (4.8-7.9)	65.20	34.80	5	8
Harandi *et al*. [42]	Kerman	ELISA	1062	77	7.2 (5.7-9.1)	2.10	8.30	-	-
Garedaghi and Bahavarnia [43]	East Azerbaijan	ELISA	1500	19	1.2 (0.7-1.9)	0.83	1.76	0.93	1.80
Heidari *et al*. [44]	Ardebil	ELISA	670	12	1.7 (0.9-3.1)	2.60	1.68	1.10	2.58
Dadkhah *et al*. [45]	East Azerbaijan	IFA	250	8	3.2 (1.3-6.3)	-	-	-	-
Rakhshanpour *et al*. [17]	Qom	ELISA	1564	25	1.5 (1.1-2.3)	2.20	0.90	2.10	1.20
Asgari *et al*. [46]	Markazi	ELISA	578	20	3.4 (2.1-5.3)	2.31	4.15	1.46	6.98
Zibaei *et al*. [47]	Lorestan	ELISA	617	95	15.3 (12.4-18.8)			5.30	38.90
Shahrokhabadi *et al*. [48]	Kerman	ELISA	486	9	1.8 (0.8-3.5)	3.10	1.94	1.30	3.25
Fallah-Omrani *et al*. [49]	Lorestan	ELISA	927	25	2.6 (1.7-3.9)	3.59	2.12	1.20	3.24
Sadjjadi *et al*. [50]	Khorasan Razavi	ELISA	1033	55	5.3 (4.1-6.9)	4	5.92	4.10	6
Manouchehri-Naeini *et al*. [34]	Chaharmahal va Bakhtiari	ELISA	1280	26	2.1 (1.3-2.9)	-	-	-	-
Ilbeigi *et al*. [12]	Isfahan	ELISA	635	7	1.1 (0.4-2.2)	2.24	0.27	1.49	-

CI=Confidence interval, ELISA=Enzyme-linked immunosorbent assay, CIE=Counter immune electrophoresis, IFA=Indirect fluorescent antibody test, IHA=Indirect hemagglutination assay

**Figure-5:**
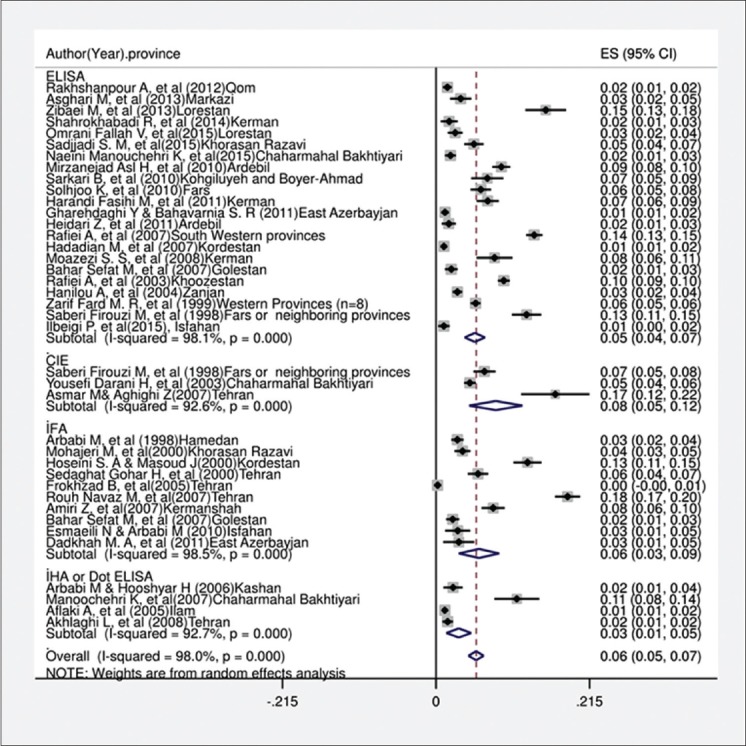
Forest plot for distribution hydatid cyst serology in terms of lab methods in Iran.

Five types of serological diagnostic assays including enzyme-linked immunosorbent assay (ELISA), indirect fluorescent antibody test (IFA), indirect hemagglutination assay (IHA), dot-ELISA, and counter immune electrophoresis (CIE) were conducted in the different studies included in this analysis. The CIE method was used in three studies, the IFA test in 10, ELISA in 22, and other serological methods in 22. A subgroup analysis of methods is shown in Figure-5 and Table-2.

**Table-2:** Subgroup meta-analysis of the prevalence of hydatid cyst serology for characteristics of the included studies.

Variable	n	Prevalence (%)	95% CI		I^2^ (%)	p-value

			Lower	Upper		
Age (year)						
<20	19	3.4	0.7	12.4	89.4	p<0.001
20-40	20	10.6	2.4	14.1	90.1	
41-60	21	7.5	4.1	13.3	88.8	
>60	16	5.4	2.7	8.4	87.8	
Sex						
Male	22	9.2	6.7	11.8	99.8	p<0.001
Female	22	8.3	6.4	10.2	99.8	
Residence						
Urban	8	3.0	1.8	4.2	88.6	p=0.048
Rural	8	6.5	4.0	9.1	96.0	
Lab method						
ELISA	22	5.4	4.0	6.9	98.0	p<0.001
CIE	3	8.4	4.7	12.1	92.8	
IFA	10	6.0	3.2	8.9	98.5	
Others*	4	3.2	1.4	4.9	92.7	
Education						
Illiterate	10	6.9	2.3	11.5	93.6	p<0.001
School	6	8.3	4.4	12.6	90.1	
Diploma	7	5.6	1.7	8.6	96	
University	7	4.3	1.5	7.1	98.4	

* IHA=Dot ELISA, CI=Confidence interval, ELISA=Enzyme-linked immunosorbent assay, CIE=Counter immune electrophoresis, IFA=Indirect fluorescent antibody test, IHA=Indirect hemagglutination assay

**Figure-6:**
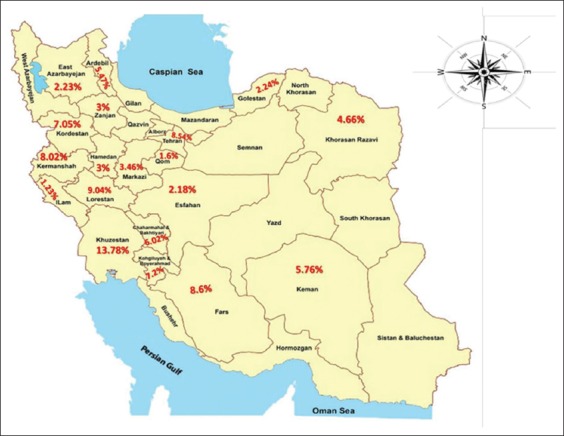
Distribution of Iranian cystic echinococcosis seroprevalences in different provinces.

The seropositivity rate of human CE infection in some provinces was determined (Figure-6). Based on available information, CE infection was more common in warm and humid climates than in colder and drier regions.

**Figure-7:**
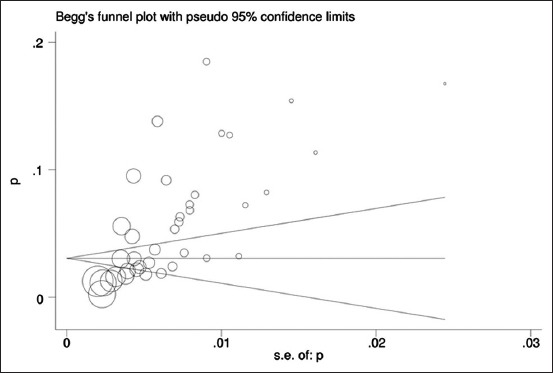
Begg’s funnel plot for assessing publication bias in the seroprevalence analysis of hydatidosis.

The seroprevalence of CE among people who had close contact with dogs, consumed raw or uncooked vegetables, farmers and housewives and at last who had low level of education were significantly higher than that of others groups. Begg’s funnel plot (Figure-7) and the Egger weighted regression test showed that there was a significant publication bias (p<0.001).

## Discussion

Since the geographical distribution of CE is worldwide, it is crucial to determine the status of CE in humans. This systematic review and meta-analysis were performed by reviewing published literature. From 40 selected studies and 49,460 participants, 3090 CE seropositive cases were identified, resulting in a CE seroprevalence in the general population of 6% (95% CI: 5.0-7.0%).

Iran has three water borders, namely the Caspian Sea, Oman Sea, and Persian Gulf. There are different geographical regions with distinct climates in Iran [51,52]. In Iran, three distinct cycles of *E. granulosus* have been identified: A domestic cycle between dogs and livestock, a desert cycle between dogs and camels, and a sylvatic cycle between wild carnivores and wild ruminants [53].

The seroprevalence of CE varies by region in Iran, as a result of differences in climate and other conditions. CE infection was found to occur more often in warm and humid climates than in colder and drier regions [14]. The highest prevalences of CE in human and animal are found in countries in temperate zones, including the Mediterranean region, southern and central Russia, Central Asia, China, Australia, some regions of South America, and northern and eastern regions of Africa [9,54,55].

The annual incidence of human CE in Europe varies between 1 and 8 per 100,000 populations, with the exceptions of Ireland, Iceland, and Denmark [2,17,56,57]. Studies on different endemic areas have determined CE seroprevalences in Peru (2.6%), Spain (3.4%), Brazil (3.5-6%), India (5-9.23%), Jordan (2.4-11.4%), China (9.5-25.5%), and Greece (up to 29%) [58-67].

Human CE has been associated with several risk factors including gender, age, residential area, climate, contact with dogs, soil exposure, livestock ownership, herding occupation, hunting, eating habits (e.g., raw or unwashed vegetables), and level of education and knowledge. Of course, when risk factors are combined, they can shape the epidemiologic pattern of the disease in that region [66,68-72].

Our meta-analysis of community-based surveys showed that males had a significantly higher seroprevalence of CE infection than females (p<0.001). This may be a result of gender roles and cultural differences in endemic regions, with men more involved in farming, hunting, and herding livestock, and in closer contact with dogs. Similar differences were seen in India [66,73].

Age is one of the major factors associated with CE, with the seroprevalence of human CE increasing with age. The development of clinical symptoms takes a long time in humans, making a determination of the true age of infection difficult. Since hydatid cysts grow slowly and immune responses to CE infection in childhood persist, long-term CE may be diagnosed in adulthood [5,56,66,73,74]. The present study showed that the age groups 0-20 and 20-40 years had the lowest and highest CE seroprevalences, respectively. Similar results were seen in Pakistan [75].

Another important risk factor is living in a rural region. The higher seroprevalence of CE in rural regions than in urban areas found in this study may be attributed to rural populations being closely associated with the *Echinococcus* lifecycle. Other factors that may be responsible for the high prevalence in rural residents include low education levels, poor economic conditions and medical services, and farming and herding livestock as main occupations. Moreover, soil contaminated by dog feces and even dust containing eggs aspirated during rural activities can be major reasons for transmission of *E. granulosus* [66,72]. In this analysis, the seroprevalence of CE infection in rural regions was found to be significantly higher (p=0.048) than that of urban areas.

Climate has an effect on the geographical distribution of CE. The dominant climate in Iran is cold and arid. Our study revealed that Khuzestan Province has the highest seroprevalence (13.78%) of CE in Iran. Khuzestan Province has high humidity and suitable temperature for the maintenance of *E. granulosus* eggs and continuation of the parasite’s lifecycle. In contrast, Qom Province has low humidity and is a semi-desert climate; thus, agriculture and husbandry are not possible; correspondingly, Qom Province had the lowest CE seroprevalence (1.6%) in Iran [17,18].

Dogs that guard livestock are an important source of *E. granulosus* infections. Interactions between humans and livestock, particularly in rural areas, as well as close contact with dogs can increase the rate of CE seroprevalence [72,76].

The prevalence of *E. granulosus* infection in definitive hosts was 19.1% in dogs, 2.3% in golden jackals, and 5% in red foxes, whereas the prevalences in intermediate hosts, namely sheep (11.1%), goats (6.3%), cattle (16.4%), and buffaloes (12.4%), in Lorestan Province have been reported [53,77]. In addition, a survey in western provinces of Iran showed that the prevalence of *E. granulosus* infection in stray dogs and red foxes was 13.25% and 4.54%, respectively [78].

A majority of dog owners, especially in rural areas, neglected to take precautions against infection such as care in feeding their dogs, maintaining the place where they kept them, proper handling of their feces, and regular medical checkups [55]. With intimate contact between children and dogs, including playing, there is the possibility of parasite transmission through accidentally swallowed eggs[79]. Eggs adhere to hairs around an infected dog’s anus and are found on the muzzle and paws. Indirect transfer of eggs either through contaminated water and uncooked infected vegetables or arthropods intermediates such as flies can also result in infection of humans [3,69]. It is crucial that slaughterhouse scraps, which may include cyst-infected livers and lung tissues, be kept away from dogs and be disposed of properly [15,53,80,81].

Antibody assays are useful serological tests to detect prior *E. granulosus* infection, based on their low cost and ease of use. However, some patients with CE do not demonstrate a detectable immune response [81,82]. According to epidemiological investigations, ELISA test was principal test used by researchers. Therefore, this could be the most important test to evaluate the relative importance of different sources of hydatidosis infection use in CE. CE serological tests have been useful in diagnosis of CE in humans, but, in terms of both specificity and sensitivity, there are remarkable differences among the various tests. An optimal test should have both high specificity and high sensitivity. Earlier CE diagnostic tests with low sensitivity and low specificity, including the Casoni intradermal test, the complement fixation test, IHA, and the latex agglutination test, have been replaced by ELISA, IFA, immunoelectrophoresis, and immunoblotting basic methods as routine tests [72,83-86].

Based on the results of this analysis, the rate of CE was determined from several studies in several regions of Iran. Seroprevalences ranged from 1.2% to 21.4% based on serological methods, mainly ELISA [14,17]. According to studies conducted between 1998 and 2007, the most commonly used serodiagnostic test was IFA. This test was used to detect CE in some areas of Iran [87].

IFA is a useful and cost-effective test, but it is difficult to perform in a routine laboratory. The sensitivity of IFA is between 82.5% and 91.6%, and the specificity is between 83% and 100% [87,88]. However, from 2007 to 2015, ELISAs have been used for CE screening. Several studies have indicated that the ELISA technique shows greater sensitivity (87.5-96.7%), specificity (89.7-100%), and 92.3% diagnostic efficacy for CE than other serological methods [81,89-94]. Moreover, ELISA allows large numbers of sample to be tested at the same time, representing a major advantage over other serological studies.

In the case of IHA, sensitivities have been found to range between 78.1 and 90%, with specificities of 93.9-97.5% [87,94,95]. However, it seems that Dot-ELISA, with a sensitivity range of 86-100% and a specificity range of 90-99.5%, has greater diagnostic value than other tests [19,96,97].

## Conclusion

This analysis synthesizes valuable information from prior studies. Our results indicated that there is a high seroprevalence of CE in the general population of Iran and that this country should be considered an endemic area of *E. granulosus* infection. This point is worthwhile to mention that ELISA is more sensitive and specificity than other immune assays in CE diagnosis, and also, the present study provides a comprehensive view of the seroepidemiology of CE in the Iranian general population. Considering the high prevalence of prior *E. granulosus* infection in the definitive and intermediate hosts and the distribution of this parasite in Iran, defining this country as endemic for CE can be justified. Due to the significance of this disease, proper preventive strategies should be considered.

## Authors’ Contributions

SG and MS conceived the idea for this review. AT and MR searched the databases for potentially eligible articles based on their titles and abstracts. SS and AD participated in the study design and wrote the manuscript. SS and SM critically reviewed the manuscript. All authors read and approved the final manuscript for publication.
